# Independent flavonoid and anthocyanin biosynthesis in the flesh of a red-fleshed table grape revealed by metabolome and transcriptome co-analysis

**DOI:** 10.1186/s12870-023-04368-8

**Published:** 2023-07-15

**Authors:** Renxiang Lu, Miaoyu Song, Zhe Wang, Yanlei Zhai, Chaoyang Hu, Avihai Perl, Huiqin Ma

**Affiliations:** 1 College of Horticulture, China Agricultural University, Beijing, 100193 China; 2 Department of Fruit Tree Sciences, Agricultural Research Organization, The Volcani Center, Bet Dagan, Israel

**Keywords:** Red-fleshed grape, Transcriptome, Metabolomics, Anthocyanin, Flavonoid

## Abstract

**Background:**

Red flesh is a desired fruit trait, but the regulation of red flesh formation in grape is not well understood. ‘Mio Red’ is a seedless table grape variety with light-red flesh and blue-purple skin. The skin color develops at veraison whereas the flesh color develops at a later stage of berry development. The flesh and skin flavonoid metabolomes and transcriptomes were analyzed.

**Results:**

A total of 161 flavonoids were identified, including 16 anthocyanins. A total of 66 flavonoids were found at significantly different levels in the flesh and skin (fold change ≥ 2 or ≤ 0.5, variable importance in projection (VIP) ≥ 1). The main anthocyanins in the flesh were pelargonidin and peonidin, and in the skin were peonidin, delphinidin, and petunidin. Transcriptome comparison revealed 57 differentially expressed structural genes of the flavonoid-metabolism pathway (log_2_fold change ≥ 1, FDR < 0.05, FPKM ≥ 1). Two differentially expressed anthocyanin synthase (ANS) genes were annotated, *ANS2* (*Vitvi02g00435*) with high expression in flesh and *ANS1* (*Vitvi11g00565*) in skin, respectively. One dihydro flavonol 4-reductase (*DFR, Vitvi18g00988*) gene was differentially expressed although high in both skin and flesh. Screened and correlation analysis of 12 ERF, 9 MYB and 3 bHLH genes. The Y1H and dual luciferase assays showed that MYBA1 highly activates the *ANS2* promoter in flesh and that ERFCBF6 was an inhibitory, EFR23 and bHLH93 may activate the *DFR* gene. These genes may be involved in the regulation of berry flesh color.

**Conclusions:**

Our study revealed that anthocyanin biosynthesis in grape flesh is independent of that in the skin. Differentially expressed *ANS*, *MYB* and *ERF* transcription factors provide new clues for the future breeding of table grapes that will provide the health benefits as red wine.

**Supplementary Information:**

The online version contains supplementary material available at 10.1186/s12870-023-04368-8.

## Background

Grape is one of the most important fruit in the world, with several thousands of recorded varieties. In 2019, grapevine planting area was 6.93 million ha, and production reached 77.14 million tons (http://www.fao.org/faostat/zh/ # data/QC). China is the largest table grape producer and consumer. Grape berry development follows a typical double-sigmoid growth curve. Veraison, the start of stage III on this curve, is marked by berry softening and changing skin color [1]. Based on the skin color, grape varieties can be divided into two major categories: red grapes, which are purple, red or pink, and white grapes, which are light green to golden-colored at ripening.

Flavonoids are a class of secondary metabolites that include flavanones, proanthocyanidins and anthocyanidins. They scavenge reactive oxygen species in plants, and are of high value for human health [2]. Anthocyanins are one of the most important flavonoids in grape berries determining berry color. Six anthocyanidins are commonly identified in grape berries: cyanidin, peonidin, delphinidin, malvidin, pelargonidin and petunidin [3]. The dominant anthocyanin differs in different grape varieties, and pelargonidin derivatives are barely detectable in most *Vitis vinifera* varieties [4].

Anthocyanin biosynthesis via the phenylpropanoid- and flavonoid-biosynthesis pathways, and the main regulatory transcription factors (TFs), have been widely elucidated in grape skin [5]. In the flavonoid-biosynthesis pathway, proteins encoded by late biosynthesis genes (LBGs) including dihydro flavonol 4-reductase (DFR), anthocyanin synthase (ANS) and UDP-glucose: flavonoid-3-*O*-glucosyltransferase (UFGT) are the most important enzymes for anthocyanin biosynthesis and stability [6]. Anthocyanins are synthesized in the cytoplasm and then transported to vacuoles for storage. However, the mechanism governing anthocyanin transport is unclear. Four types of anthocyanin transporters have been reported in grape: glutathione S-transferase (GST), ATP-binding cassette (ABC) transporters, multidrug and toxic compound extrusion transporters (MATE), and bilitranslocase (BTL) [7]. A total of 161 transporters were identified in a study of the grape vacuolar proteome in our laboratory, among which 13 ABC transporters were more abundant at fruit maturity [8].

Among the large MYB TF family, subgroups 5 and 6 are regarded as inhibitors and activators of anthocyanin biosynthesis, respectively [9]. *V. vinifera* (Vv) MYBA1 and VvMYBA2 have been identified as the main TFs regulating grape-skin coloring [10, 11]. Subgroup III of basic helix-loop-helix (bHLH) has been reported to be involved in anthocyanin biosynthesis [12, 13]; VvMYC1 interacts with MYBs in grapes and participates in the regulation of anthocyanin and proanthocyanin biosynthesis [14]. Some ethylene response factor (ERF) TFs have also been found to regulate anthocyanin accumulation in *Arabidopsis thaliana* [15], as well as apple [16] and other fruit.

The differential expression of key enzyme genes regulated by TFs in various developmental periods and organs of plants gives tissue synthesis specificity to anthocyanins, especially in some red-fleshed fruit types [17]. In apple, MdMYB1 binds to the *MdDFR* and *MdUFGT* promoters, and MdMYBA regulates the expression of *MdANS*, which is involved in the synthesis of anthocyanin in the pericarp [18]. *MdMYB10* regulates the synthesis of anthocyanin in apple pulp, it interacts with MdbHLH3/33 to increase the activity of the *MdDFR* promoter [19]. Genotypic differences among varieties lead to different key structural genes for anthocyanin synthesis in red kiwifruit, AcUFGT3a is the key synthetase for anthocyanin in ‘Hongyang’ fruit, *LDOX*, *AcF3GT1*, and *AcF3GGT1* control key structural genes for anthocyanin accumulation in some red-fleshed fruits of Chinese kiwifruit, respectively [20]. AcMYB110 has been shown to activate *F3GT* expression, and AcMYB10 interacts with AcbHLH42 to activate the expression of *LDOX* and *F3GT* [21].

For most red grape varieties, anthocyanin only exists in vacuoles of the 3–4 layers of the skin, and the flesh and juice are white. Red flesh is rare among grape varieties, with ‘Alicante Bouschet’ being the best-known of these: it has rose-red flesh, and is used as a teinturier variety to enhance the color of red wine. ‘Yan73’ is a Chinese teinturier variety from a cross between ‘Alicante Bouschet’ and ‘Muscat Hamburg’ [22]. In ‘Yan73’, anthocyanin appears to accumulate in the flesh before veraison, and in comparison to its dark-skinned, white-fleshed parent ‘Muscat Hamburg’, the accumulation of anthocyanins in ‘YAN73’ flesh likely due to the tissue-specific expression of *OMT*, *AM3*, *GST*, *F3’5’H*, *LDOX* and *MYBA1* genes in flesh tissue [23].

The red-flesh phenotype exists in many fruits, such as orange, kiwi, apple and plum, among others. With their higher antioxidant capacity and attractive color, red-fleshed fruit are welcomed by the market. Our laboratory bred a red-fleshed table grape—variety ‘Mio Red’. This grape is destined for fresh consumption, but it is also suitable for making juice. Comparing and analyzing the different coloring in the flesh and skin of ‘Mio Red’ berries could provide a further understanding of the coloring regulation in berry flesh, which could in turn benefit future breeding of such grapes as healthy snacks.

## Results

### Flavonoids in the skin and flesh of ‘Mio Red’ at ripening

**Fig. 1:**
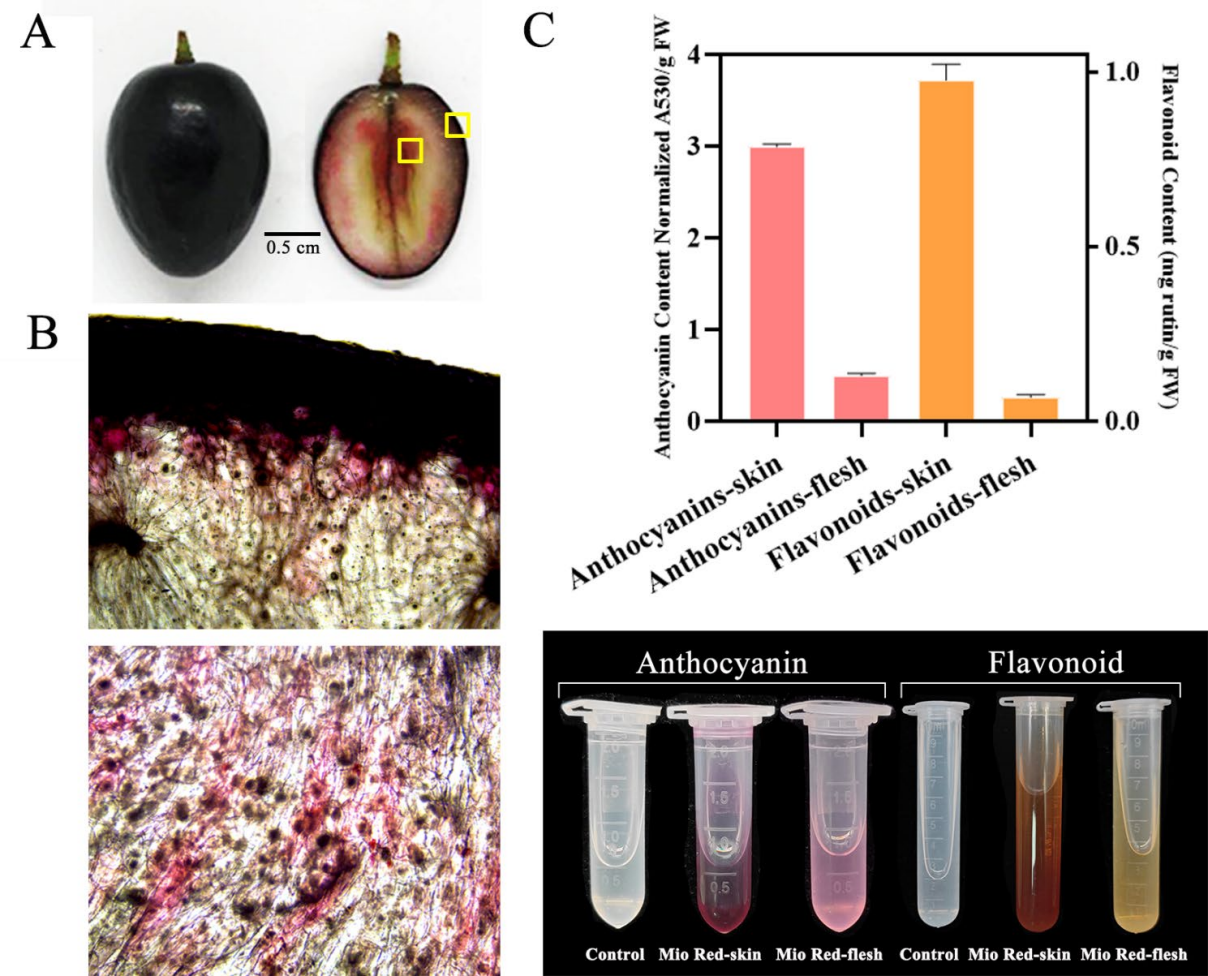
Anthocyanin accumulation and total flavonoids in the skin and flesh of ‘Mio Red’. **(A)** Colored skin and flesh at berry ripening (86 DAA). **(B)** Distribution of anthocyanin in the flesh. Upper figure: observation of skin and flesh. Lower figure: flesh near the style. **(C)** Content of anthocyanin and total flavonoids in the skin and flesh; significance between groups was determined by Student’s *t-*test, different lowercase letters indicate significant differences. Error bar indicates standard error, *p* < 0.05

The skin and flesh of ‘Mio Red’ berry demonstrated different colors at ripening. The skin was uniformly deep purple–black, while the flesh color was red with a higher concentration around the style (Fig. 1A). Microscopic observation supported flesh coloration as an independent event, rather than due to diffusion of anthocyanin from the skin, because layers of non-colored cells could be seen between the skin and the colored flesh tissue, and in the flesh, the colored areas were concentrated in a multiple-subarea pattern (Fig. 1B). The anthocyanin content of ‘Mio Red’ flesh was about 6.2 times lower than that of the skin. Total flavonoid content demonstrated the same trend: in the flesh it was 14.25 times lower than in the skin (Fig. 1C).

**Table 1:** Anthocyanins identified in skin and flesh of ‘Mio Red’ grape

Metabolite	Content		VIP	LogFC
	Skin	Flesh		
Peonidin *O*-hexoside	2.25E + 08	3.55E + 07	0.6	‒2.66
Peonidin 3-*O*-glucoside	2.19E + 08	2.82E + 07	0.63	‒2.95
Peonidin 3,5-diglucoside	2.27E + 07	1.91E + 06	0.7	‒3.57
Peonidin*	9.38E + 06	N.D.	1.65	‒19.99
Delphinidin 3-*O*-glucoside*	2.38E + 08	1.51E + 06	1.01	‒7.3
Delphin	1.40E + 08	2.04E + 06	0.91	‒6.1
Petunidin 3-*O*-glucoside	2.39E + 08	1.95E + 06	0.98	‒6.94
Petunidin 3,5-diglucoside*	2.89E + 07	1.06E + 05	1.05	‒8.09
Cyanidin 3-*O*-glucoside	4.40E + 07	9.01E + 06	0.56	‒2.29
Cyanidin 3-*O*-galactoside	3.74E + 07	7.55E + 06	0.56	‒2.31
Cyanidin 3,5-*O*-diglucoside	8.89E + 06	1.26E + 06	0.61	‒2.82
Cyanidin 3-*O*-rutinoside	2.40E + 06	2.01E + 05	0.7	‒3.58
Pelargonin	6.51E + 06	3.17E + 05	0.77	‒4.36
Pelargonidin	2.94E + 04	5.22E + 04	0.44	0.83
Pelargonidin 3-*O*-*β*-*D*-glucoside	6.63E + 07	8.91E + 07	0.24	0.43
Malvidin 3,5-diglucoside	4.71E + 06	4.16E + 05	0.69	‒3.5

Three anthocyanins with star (*) were DAMs [fold change (FC) ≥ 2, FC ≤ 0. 5, VIP ≥ 1]. N.D. = not detected

A total of 161 flavonoids were identified in the colored skin and flesh of ‘Mio Red’, which could be further divided into 8 groups of 57 flavones, 30 flavonols, 14 flavanones, 17 polyphenols, 16 anthocyanins, 19 flavonoids, 5 isoflavones and 3 proanthocyanins (Table S2). Clustering analysis of the flavonoid data revealed obvious differences between and within the two samples (Fig. S1A). Principal component analysis showed a clear trend of separation between the sample groups, with PC1 and PC2 contributing 84.23% and 5.89% of the difference, respectively (Fig. S1B), indicating that there was a significant difference between the metabolites in the skin and flesh of ‘Mio Red’ grape. A hierarchical heatmap clustered all biological replicates together, and metabolites of the same type together (Fig. S1C), demonstrating the high reliability of the flavonoid metabolome results.

Sixty-six metabolites showed different contents in ‘Mio Red’ flesh and skin by VIP ≥ 1 and fold change ≥ 2 or ≤ 0.5. (Table S3). Compared to the skin, 63 flavonoids had a lower content, and 3 had significantly higher content in the flesh; the latter 3 were epicatechin-epiafzelechin, apigenin 6,8-*C*-diglucoside and quercetin *O*-glucoside/hesperetin 5-*O*-glucoside. These three flavonoids were barely detected in the skin (Fig. S2A). The six common anthocyanins reported in grape were detected in both the skin and flesh of ‘Mio Red’, but their contents were significantly different in the two tissues. There were three differentially accumulated anthocyanins in the skin and flesh: peonidin, delphinidin 3-*O*-glucoside, petunidin 3, 5-diglucoside (Table 1). Peonidin derivatives were the main anthocyanin in the skin, whereas the flesh was dominated by pelargonidin derivatives (Table S4), further supporting the independent coloration of flesh and skin of ‘Mio Red’.

Four flavonoid-biosynthesis pathways with significant differences between the flesh and skin were found by locating the differentially accumulated metabolites (DAMs) in the KEGG pathway; the flavonoid and flavonol biosynthesis pathway had the largest number of metabolites showing differential levels, followed by the flavonoid biosynthesis pathway (Fig. S2B).

### Transcriptome differences between ‘mio red’ skin and flesh

**Fig. 2:**
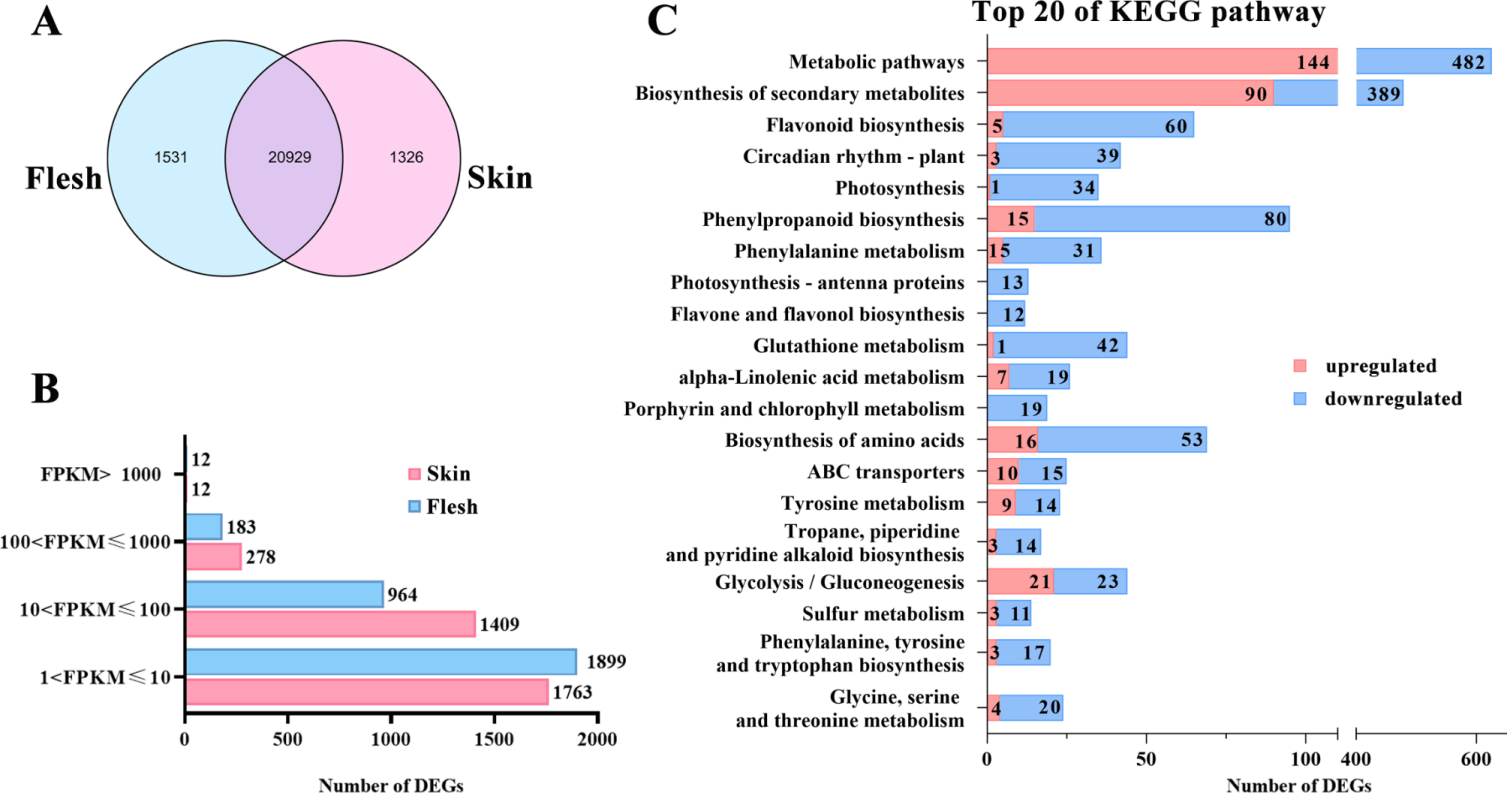
Transcriptome analysis of the skin and flesh of ‘Mio Red’ grape 86 DAA. **(A)** Venn diagram of all expressed genes in the skin and flesh. **(B)** FPKM value distribution of DEGs (log2fold change ≥ 1, FDR < 0.05, FPKM ≥ 1) in the skin and flesh. **(C)** Enriched KEGG pathways (top 20) of DEGs

Three biological replicates of each sample (skin and flesh of ‘Mio Red’ grape berries 86 DAA) were subjected to RNA-seq analysis. After filtering, transcriptome sequencing gave 149,774,478 and 158,097,474 clean reads from skin and flesh respectively, of which 93.8% reached Q30 (Table S5), and 78.01–83.80% were uniquely matched with the grape reference genome (map quality ≥ 30) (Table S6). There were 20,929 genes expressed in both skin and flesh, and 1,326 and 1,531 genes were specifically expressed in the skin and flesh, respectively (Fig. 2A).

With |log_2_fold change| ≥ 1, FDR < 0.05 and FPKM ≥ 1 as thresholds, 3,970 DEGs were screened from the transcriptome data (Table S7), among which 1,420 genes were upregulated and 2,550 genes downregulated in the flesh compared to the skin. Expression-level analysis showed that about 0.2% of the DEGs were very highly expressed (FPKM > 1000), 5.4% were highly expressed (FPKM ~ 100–1000), and 56.2% had FPKM of 1–100 (Fig. 2B). Further KEGG-pathway enrichment revealed that metabolic pathway, flavonoid biosynthesis pathway, phenylpropanoid biosynthesis, and flavonoid and flavonol biosynthesis pathway were among the top-ranked, in addition to the enrichment of genes encoding ABC transporter protein (Fig. 2C).

### Expression of structural genes of flavonoid- and anthocyanin-biosynthesis pathways

**Fig. 3:**
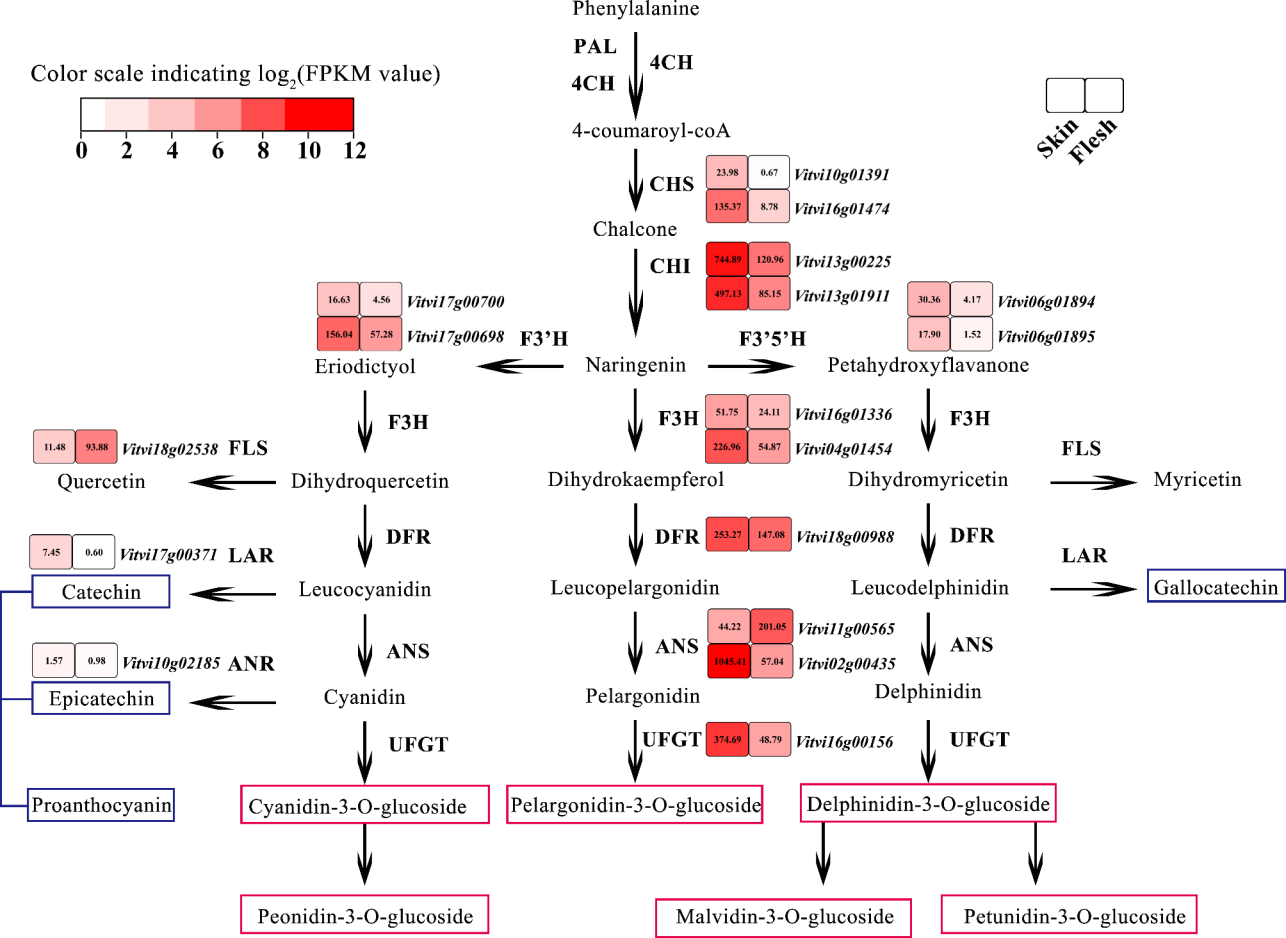
Expression profiles of DEGs involved in flavonoid-biosynthesis pathway. Scale from white (low) to red (high) indicates FPKM value in skin and flesh of ‘Mio Red’ 86 DAA. Red and blue boxes indicate anthocyanins and polyphenols, respectively

A total of 2 *CHS*, 2 *CHI*, 2 *F3’H*, 2 *F3’5’H* and 2 *F3H* genes were identified as DEGs in flavonoid pathway, among which, these genes were down-regulated in flesh, consistent with the low content of total flavonoids in flesh tissue. (Table S8). Among *F3H, F3’H* and *F3’5’H*, *F3H* had the highest FPKM value, followed by *F3’H*, and *F3’5’H* with the lowest FPKM; one *F3’5’H* (*Vitvi06g01895*) exhibited the highest fold change in transcript abundance, with FPKM value in the skin that was about 12-fold higher than in the flesh. The significant difference in *F3’5’H* expression may be lead to the difference in anthocyanin ratio between delphinidin 3-*O*-glucoside and petunidin 3,5-diglucoside (Fig. 3).

Only one *DFR* gene was differentially expressed, although its expression was high in both skin and flesh. Two differentially expressed *ANS* genes were identified: *ANS1* (*Vitvi02g00435*) and *ANS2* (*Vitvi11g00565*) were highly expressed in the skin and flesh, respectively. The FPKM value of *ANS2* was 201 in the flesh, 5 times that in the skin. Only one DEG was revealed with *UFGT*, with about 8 times higher expression in the skin than in the flesh (Fig. 3).

The transcript abundance of genes involved in flavonol and proanthocyanidin biosynthesis was significantly lower than that of the anthocyanin-biosynthesis branch. It is worth noting that the expression level of *FLS* (*Vitvi18g02538*) in the flesh was 8.2 times higher than that in the skin, which is in agreement with the metabolome result that the flesh of ‘Mio Red’ tends to accumulate more flavonols relative to the skin. DEGs *LAR* and *ANR*, which had extremely low expression in both the skin and flesh, were consistent with the detected low proanthocyanidin content.

### Flavonoid-biosynthesis gene expression during berry development

**Fig. 4:**
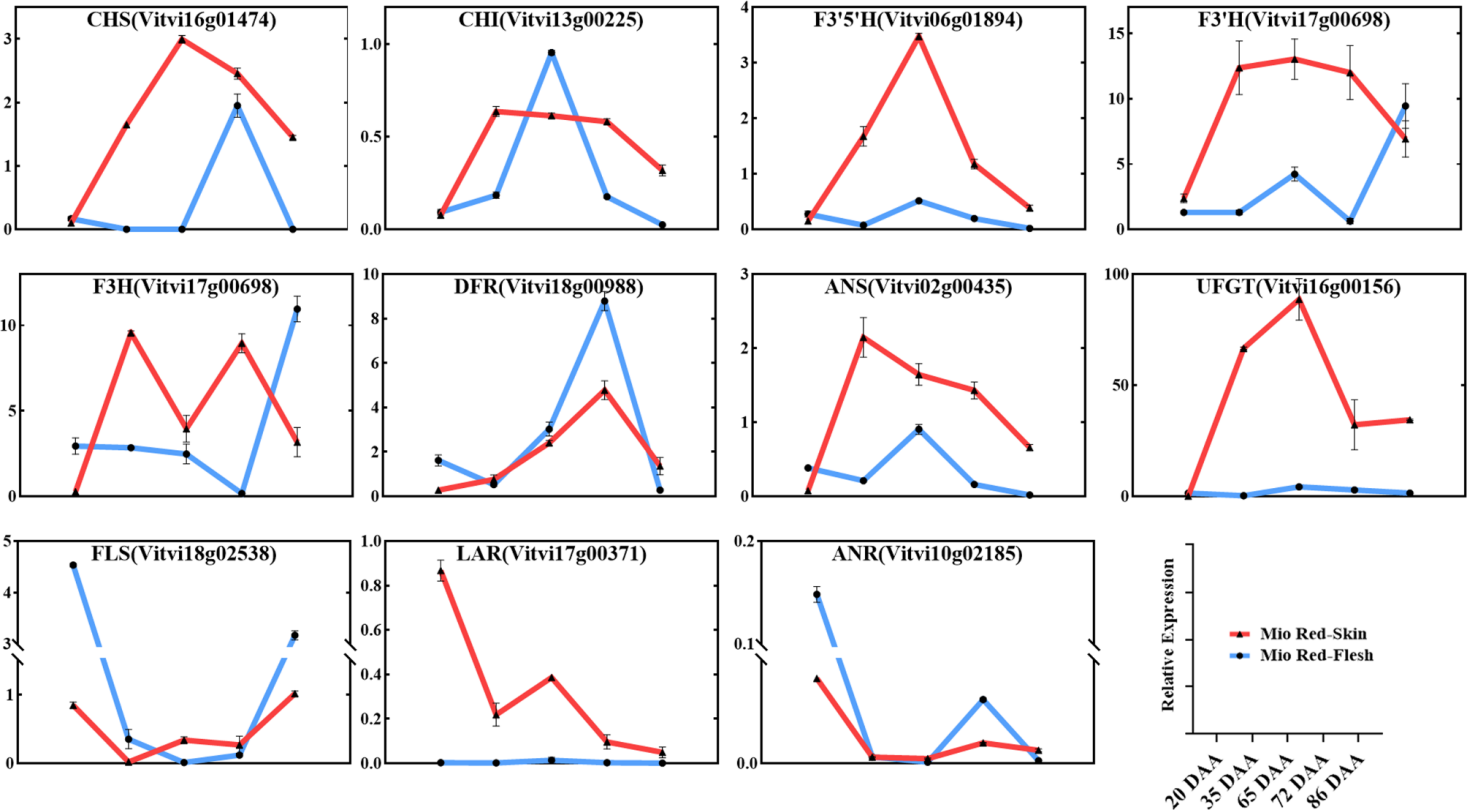
QRT-PCR of genes associated with flavonoid metabolism during ‘Mio Red’ grape berry development. All qRT-PCR calculations of expression levels were normalized using the corresponding Ct values of the grape β-actin gene. Values are means ± SE of three biological replicates, *p* < 0.05

The expression of structural flavonoid-biosynthesis genes was found to align with the specific flavonoid and anthocyanin accumulation characteristics in the flesh and skin. In the phenylpropanoid pathway, *CHS* and *CHI* demonstrated overall increased expression in the early stage of berry development, which then decreased from veraison to berry ripening. Overall higher expression was found in the skin than in the flesh, in agreement with the higher flavonoid and anthocyanin contents in the skin. High expression of *CHS* and *CHI* in the flesh was found on 72 and 65 DAA, respectively (Fig. 4).

In the flavonoid pathway, *F3’5’H*, *F3’H*, *F3H*, *ANS*, *UFGT* and *LAR* showed overall higher expression in the skin than in the flesh, especially *UFGT* which demonstrated the largest difference in expression. *F3’H* and *F3H* revealed higher expression in the flesh than in the skin 86 DAA, supporting the late anthocyanin accumulation feature of ‘Mio Red’ flesh. Moreover, expression of *FLS* and *ANR*, key genes for the biosynthesis of flavonols and proanthocyanins, respectively, had generally higher expression in the flesh than in the skin (Fig. 4), supporting the high levels of apigenin 6,8-*C*-diglucoside, epicatechin-epiafzelechin and quercetin *O*-glucoside/hesperetin 5-*O*-glucoside found in the flesh.

### Identification and correlation analysis of TFs related to flavonoid biosynthesis

**Fig. 5:**
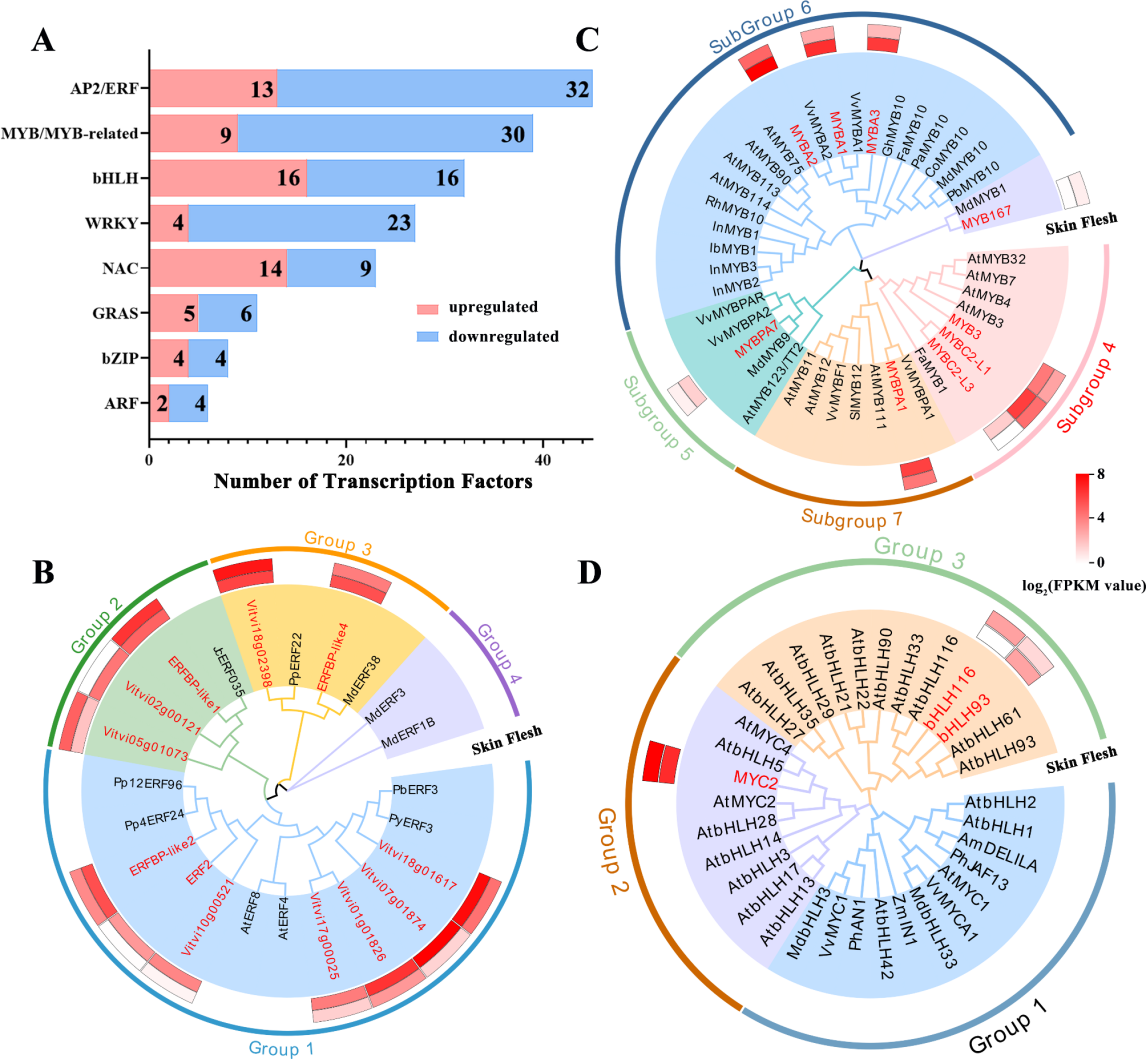
Clustering of differentially expressed TFs in the skin and flesh of ‘Mio Red’. **(A)** Expression profile of major TF families. Red and blue colors indicate the number of TFs that are up- and downregulated in flesh compared to skin, respectively. **(B)** Phylogenetic tree analysis of ERF DEGs. **(C)** Phylogenetic tree analysis of R2R3-MYB DEGs in subgroups 4/5/6/7. **(D)** Phylogenetic tree analysis of bHLH DEGs in subgroup III. Scale from white (low) to red (high) indicates FPKM value. GenBank accession numbers of the TFs are listed in Table S9

The important TF families associated with fruit growth and development were identified in the transcriptomes of the skin and flesh of ‘Mio Red’, including 45 AP2/ERF, 39 MYB (including MYB-related TFs), 32 bHLH, 27 WRKY and 23 NAC. Most of the of NAC TFs were upregulated in the flesh. The number of upregulated and downregulated genes of bHLH was the same. Members of the rest of the TF families were mostly downregulated in the flesh vs. skin (Fig. 5A).

The ERF, MYB (subgroups 4/5/6/7) and bHLH (Subgroup III) TFs annotated in the transcriptome of ‘Mio Red’ as related to the biosynthesis of anthocyanin were constructed into their respective phylogenetic trees to screen for TFs that might be involved in the regulation of berry flesh coloration. A total of 12 ERF, 9 MYB and 3 bHLH genes were screened. ERF *Vitvi18g01617* and ERFBP-like1 *Vitvi18g02398* were highly expressed in both skin and flesh. They clustered with PbERF3 of pear, JcERF035 of physic nut (*Jatropha curcas* L.) and MdERF38 of apple, respectively (Fig. 5B).

Among the MYB TFs, MYBA1/2/3 clustered in subgroup 6, and were highly expressed in both skin and flesh; these TFs may directly regulate fruit coloring. MYBC2-L3 and MYB3 were clustered in subgroup 4, which may play a role in feedback regulation and inhibit the excessive accumulation of anthocyanin. MYBPA7 was clustered in subgroup 5; its expression level was low, consistent with the low content of proanthocyanin in ‘Mio Red’ fruit. MYBPA1 was clustered in subgroup 7, homologous to VvMYBPA1 and VvMYBF1 (Fig. 5C). Among bHLH TFs, *MYC2* gene had a high expression level in both skin and flesh and clustered with AtMYC2/4/5 of *Arabidopsis thaliana*. bHLH93 and bHLH116, which clustered together with AtbHLH33, were highly expressed in the skin and flesh, respectively (Fig. 5D).

### Integrated analysis of DEGs and DAMs related to flavonoid biosynthesis and transport

**Fig. 6:**
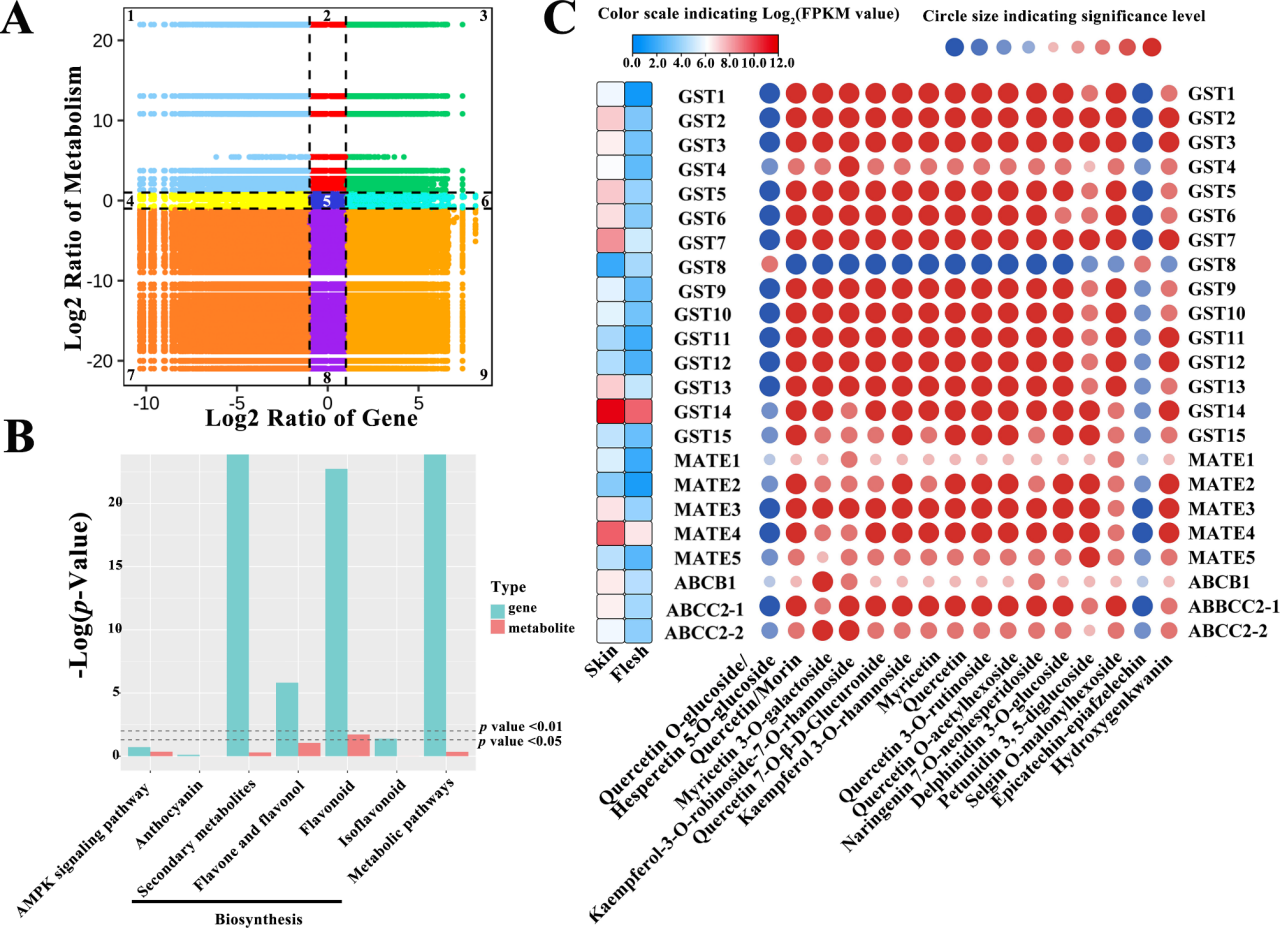
Integral analysis of DAMs and DEGs. **(A)** Nine-quadrant diagram, DEGs and DAMs with Pearson correlation coefficients greater than 0.8 were divided into 1–9 quadrants from left to right and from top to bottom with black dotted lines. **(B)** KEGG enrichment histogram. X-axis represents metabolic pathways. Y-axis represents expression as -log (*p*-value). Green and red represent DEGs and DAMs, respectively. **(C)** Expression profile of flavonoid-associated transporters and their correlation heatmap with DAMs. Red, positive correlation; blue, negative correlation; circle size indicates significance: the larger the circle, the higher the significance

The genes and metabolites with high expression/abundance in the flavonoid pathway and a Pearson correlation coefficient greater than 0.8 were selected for correlation analysis. Genes and metabolites were negatively correlated in quadrants 1 and 9, and positively correlated in quadrants 3 and 7 (Fig. 6A). The number of genes and metabolites in quadrants 7 and 9 was much higher than that in quadrants 3 and 1, indicating that genes and metabolites related to flavonoids in the flesh were significantly downregulated compared to the skin, regardless of whether the genes were positively or negatively regulated. KEGG enrichment of combined DEGs and DAMs showed that only the flavonoid-biosynthesis pathway was significantly enriched (*p* < 0.05) (Fig. 6B).

A correlation network was constructed with genes and metabolites that were enriched in flavonoid, flavone and flavonol, and anthocyanin biosynthesis, with a correlation coefficient higher than 0.8. Most genes correlated with flavonoid, and flavone and flavonol biosynthesis. Three screened *UFGT*s correlated with anthocyanin biosynthesis, and could play a key role in the differential coloring of flesh and skin (Fig. S3).

Three types of putative flavonoid-related transporters were identified: 15 GSTs, 5 MATEs and 3 ABC transporters. These may implement the transport of anthocyanins, proanthocyanins and flavonols. The transcriptome results showed that the expression levels of all of the transporter genes were higher in the skin than in the flesh. Among the transporters, *GST14* and *MATE4* had high FPKM in both skin and flesh. Correlation analysis with metabolites showed that most of the transporters were positively correlated with DAMs. Interestingly, transporter GST8 was negatively correlated with most flavonoids, including two anthocyanins: delphinidin 3-*O*-glucoside and petunidin 3,5-diglucoside, but only positively correlated with quercetin *O*-glucoside/hesperetin 5-*O*-glucoside and epicatechin-epiafzelechin (Fig. 6C).

### Effect of candidate TFs on *VvDFR* and *VvANS2* promoter activity

**Fig. 7:**
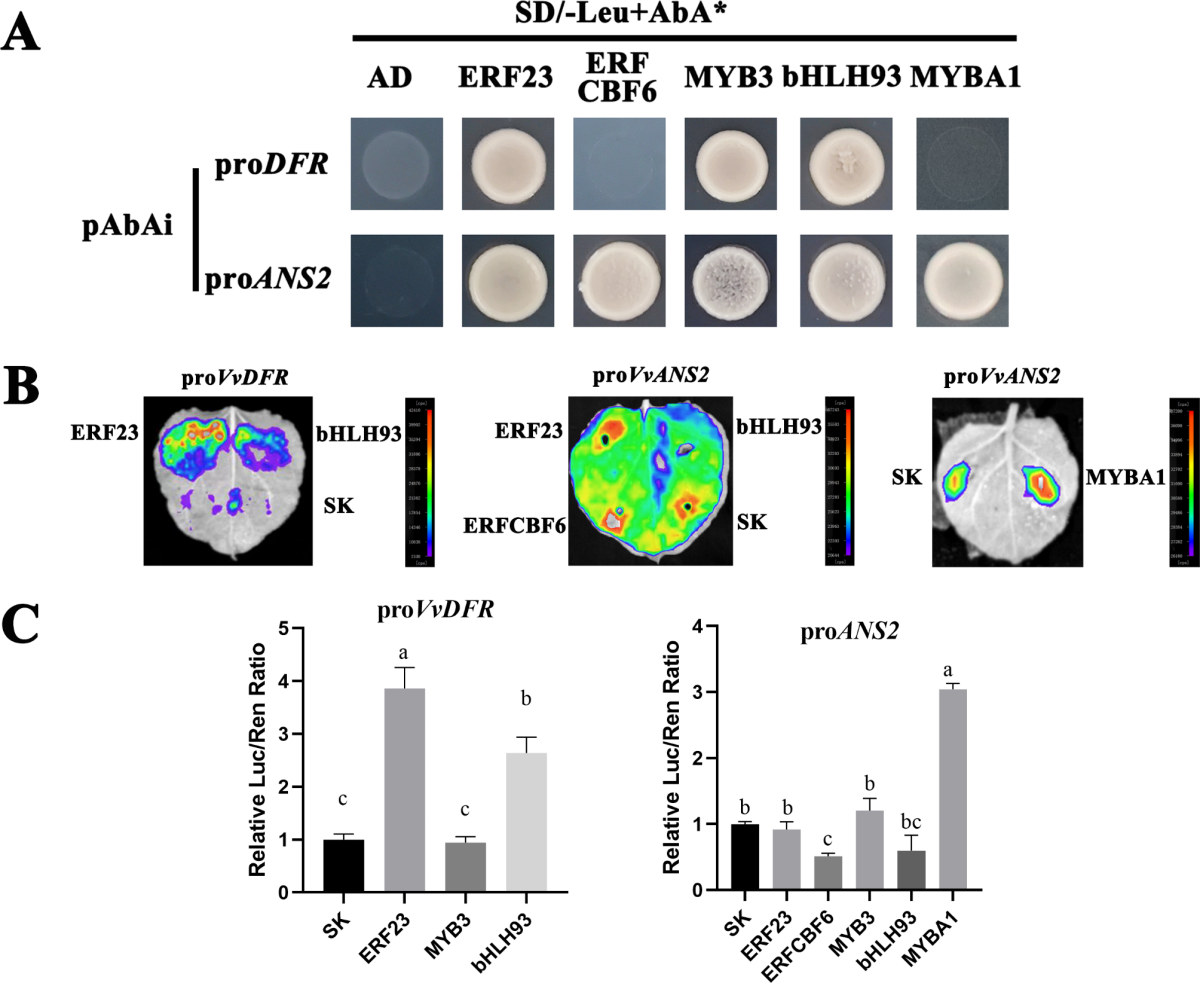
Yeast one-hybrid (Y1H) and dual luciferase (LUC) assays verifying that the candidate TFs increased or decreased the *VvDFR* and *VvANS2* promoter activity. (A) The results of Y1H. (B, C) The results of LUC assays. The promoter activity is described by the LUC/REN ratio. The lowercase letters indicate significance difference between different groups. All the data are means ± SEs of three biological replicates, *p* < 0.05

In the biological process of anthocyanin biosynthesis in grape, the downstream anthocyanin biosynthesis branch is the key to controlling color accumulation. We selected two genes encoding key enzymes of the anthocyanin synthesis pathway, *VvDFR* and *VvANS2*, which are highly expressed in flesh, as target genes to verify the regulatory role of upstream TFs. The ERFs, MYBs and bHLHs screened for possible involvement in color accumulation of ‘Mio red’ were the main candidate regulators. The promoter sequences of *VvDFR* and *VvANS2* were used as the baits, and the candidate TFs were used as preys. The Y1H assay resulted that ERF23 (Vitvi18g02398), MYB3, and bHLH93 could bind to promoters of *VvDFR* and *VvANS2*. ERFCBF6 (Vitvi02g00407) only had interaction with the *ANS2* promoter (Fig. 7A). In-vivo dual luciferase assay was conducted in tobacco leaves to detect the specific regulation of *VvDFR* and *VvANS2* gene expression by EFR23, bHLH93 and ERFCBF6. The results showed that ERF23 and bHLH23 could activate the expression of *DFR* by physically combing with the promoter region, and the activation capacity of ERF23 was significantly stronger than that of bHLH93 (Fig. 7B, left, Fig. 7C). MYB3 had no significant activation or inhibition on both *ANS2* and *DFR* (Fig. S3). MYBA1 plays an important role in the anthocyanin accumulation of grape skins, while *ANS2* may be the key structural gene that promotes the coloration of ‘Mio Red’ flesh. The dual luciferase assay results of MYBA1 and *ANS2* showed that MYBA1 could significantly activate the activity of *ANS2* (Fig. 7B right, Fig. 7C), indicating that MYBA1 also played an important role in ‘Mio red’ flesh coloring. The Luc/Ren ratio of ERFCBF6 + pro*ANS2* was decreased compared to the control, suggested slightly inhibitory effects on *ANS2* (Fig. 7B middle, Fig. 7C).

## Discussion

### Anthocyanin accumulation in the flesh of different red-fleshed grape varieties

Most colored grapes only accumulate anthocyanin in the skin at berry ripening; veraison is the common starting point for skin anthocyanin accumulation. However, the timing of anthocyanin development in the flesh is remarkably diverse among the small population of red-fleshed varieties. Flesh coloring of the teinturier wine variety Yan73 begins at stage II of the double-sigmoid curve, clearly earlier than the skin coloring [24]. ‘Summer Black’ is a table grape variety with a *V. vinifera* and *V. labrusca* hybrid background. The flesh and skin of the ‘Summer Black’ red-fleshed mutant begin to accumulate color at almost the same time as berry ripening [25]. In the case of ‘Mio Red’, coloration of the flesh occurs significantly later than that of the skin. Since flesh coloration occurs when the skin is already dark, it may have a non-light-dependent character.

The anthocyanin content in the skin and flesh of ‘Yan 73’ had little difference, and the proportion of different anthocyanin derivatives was similar, with malvidin and peonidin derivatives predominating [26]. The anthocyanin species in the skin and flesh of ‘Summer Black’ fruits were also similar, and the red flesh trait is considered to be associated with enhanced anthocyanin biosynthesis in the whole berry [24]. The significant difference between more than one-third of the flavonoid metabolites in the skin and flesh of ‘Mio Red’ grapes, including flavonols, flavonoids and anthocyanins.

### Anthocyanin biosynthesis in grape flesh is independent in ‘Mio red’

In other red-fleshed grape varieties, there is only a small difference in the type and content of flavonoids including anthocyanins in skins and flesh. However, unlike them, ‘Mio Red’ grape with peonidin derivatives dominating the anthocyanins in the skin and pelargonidin derivatives in the flesh, implying a difference in the pathways of flavonoids synthesized in the skin and flesh, may be dominated by several different key enzymes.

The differentially expressed genes encoding early synthesis enzymes of the flavonoid pathway, including *CHS*, *CHI*, *F3’H*, *F3’5’H*, and *F3H*, all showed high expression in the skin and low expression in the flesh of ‘Mio Red’ grapes. *F3’H* and *F3’5’H* produce quercetin and myricetin, respectively, in response to the b-ring hydroxylation of naringin, and these hydroxylases are required for the production of cyanidin and delphindin [27]. The significant difference in *F3’5’H* expression ultimately leads to differences in the metabolism of delphinidin and petunidin anthocyanins and is a key factor in the blue-purple coloration [28]. The specific high expression of *F3’H* and *F3’5’H* in the skin of ‘Mio Red’ corresponded to a significant accumulation of Delphinidin 3-O-glucoside.

**Fig. 8:**
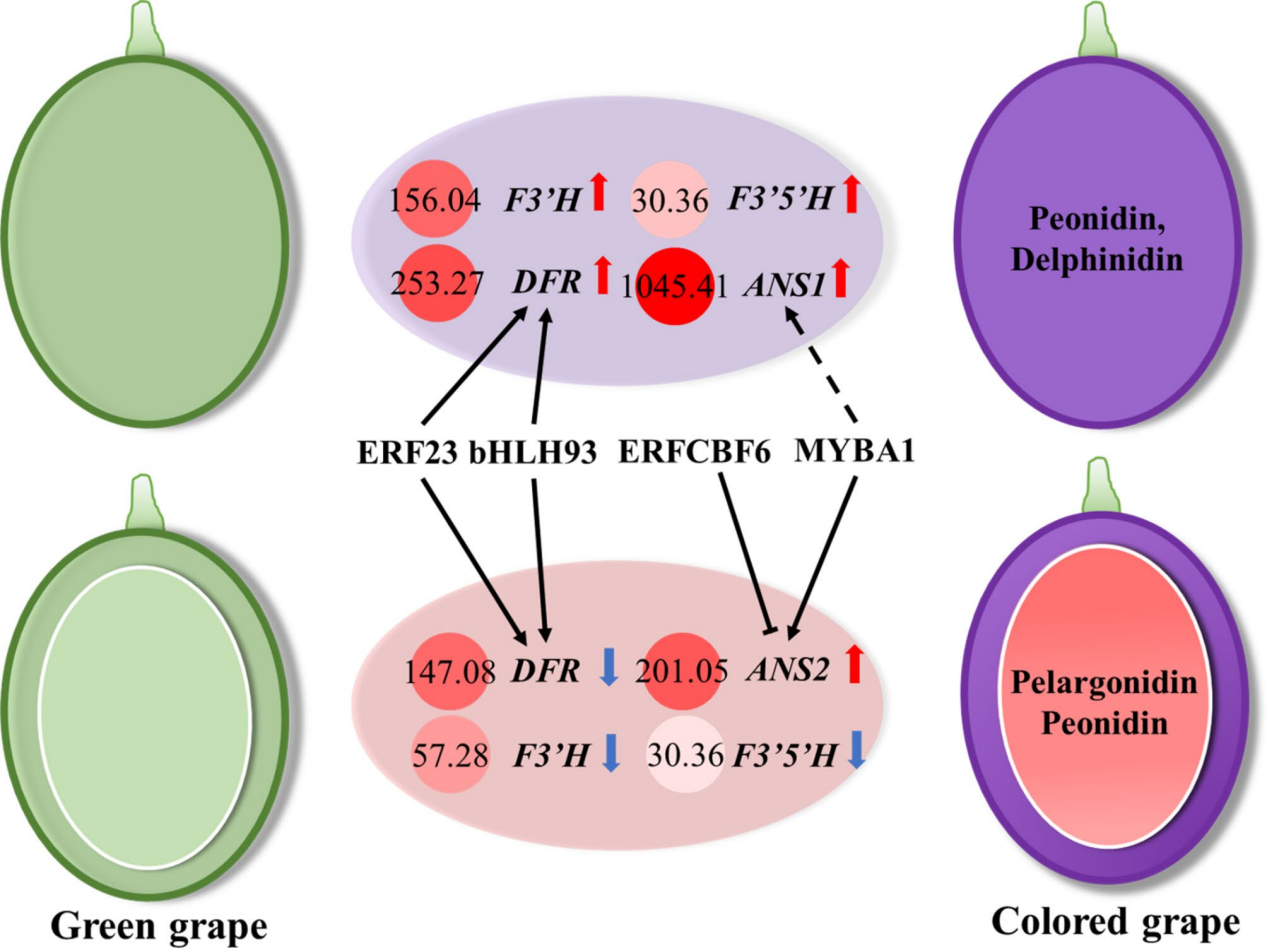
Proposed model of the regulatory mechanisms difference of coloration in skin and flesh of ‘Mio Red’. The upper indicates that the skin of ‘Mio red’ grape changes from green to red during ripening, while the lower part for the flesh

Grapes were previously considered to be devoid of pelargonidin [29], because the DFR in the studied varieties could not effectively reduce dihydrokaempferol to produce leucopelargonidin [30]. Later, an extremely low content of pelargonidin was detected in *V. vinifera* varieties such as Cabernet Sauvignon and Pinot Noir. In ‘Yan73’ grapes, DFR can complete the reduction of dihydrokaempferol, but it favors the synthesis of cyanidin and delphinidin using dihydroquercetin and dihydromyricetin as substrates, so the content of pelargonidin derivatives in ‘Yan73’ is also low [31]. However, in ‘Mio Red’ grapes, and especially in the flesh the *DFR* expression was also at a high level, pelargonidin accumulated to easily detectable amounts (Fig. 8).

Previous studies have reported that the expression pattern of *ANS* varies in different grape varieties; ANS protein accumulates in various tissues of grapevine and exhibits different effects on secondary metabolites [32, 33]. In this study, two *ANS* genes were highly expressed in the skin and flesh of ‘Mio Red’, respectively. The specific spatiotemporal expression of the two *ANS* genes may be involved in the different coloration initiation and anthocyanin content in the skin and flesh of this grape. That is, in ‘Mio Red’ grapes, flavonoid synthesis in the skin and flesh is via different synthetic pathways. Among them, *ANS1* and *ANS2* in the anthocyanin synthesis branch may be respectively the key enzyme genes dominating in the skin and flesh (Fig. 8).

### TFs regulating flavonoid accumulation

There have only been a few studies on TFs that regulate flavonoid synthesis in grape flesh. The study with ‘Yan73’ suggested that anthocyanin accumulation in the flesh tissue is probably coordinated and regulated by transcription activator VvMYBA1 and transcription repressor VvMYBC2-L1 [34]. Comparing the metabolic and transcriptomic profiles of the skin and flesh of three table grape varieties (Kyoho, Wink and Italia), it was found that besides anthocyanin, flavonol and flavanol also differ widely in the two tissues, and MYB24, MADS5 and two ubiquitin proteins (RHA2) were suggested as promising candidates for regulation of flavonoid biosynthesis in grape [35].

In the present study, we screened for TFs related to flavonoid biosynthesis, and a high level of AP2/ERF was found. The direct relationship between ERFs and flavonoid biosynthesis is still unclear. ERFs generally regulate anthocyanin accumulation in fruit by interacting with MYB or bHLH TFs [36–38].

MYBA1, MYBA2 and MYBA3 play an activating role in grape anthocyanin biosynthesis by binding to the *UFGT* promoter [39, 40]. In ‘Mio Red’, the expression of these 3 activators in the skin was significantly higher than that in the flesh. Their expression patterns in the flesh of ‘Mio Red’ could be important to its coloration. In this study, MYBA1 can significantly activate the expression of *ANS2*, which means that MYBA1 plays an important role in grape flesh coloring, besides in the skin (Fig. 8). According to the object of inhibition, suppressors which fine-tune the synthesis process and maintain fruit homeostasis [41] can be divided into two types: MYB4 and MYBC2-L1 homologs (low-molecular-weight phenolic and flavonoid, respectively) [42]. MYBC2-L1 and MYB3, screened in this study, may inhibit the accumulation of flavonoids. Although the Y1H assay showed that MYB3 could interact with the promoters of *ANS2* and *DFR*, there was no obvious inhibitory effect on their expression, and its specific action and mechanism still need to be further explored.

The MYBPA7, regulating proanthocyanin biosynthesis [43], was clustered into subgroup 5. The low expression of *MYBPA7*, especially in the flesh, could be the main reason for the low accumulation of proanthocyanins in ‘Mio Red’. Studies have shown that VvMYBPA1 activating promoters of several flavonoid-pathway structural genes, including *CHS*, *F3’5’H*, *ANS*, *LAR* and *ANR*, participates in the biosynthesis of procyanidins [44, 45]. MYBPA1 in ‘Mio red’ may mainly regulate the flavonol biosynthesis.

The bHLH93 screened from the phylogenetic tree were clustered together with AtbHLH116 (ICE1) and AtbHLH93 (ICE2), which reported as a role in low-temperature stress [46]. In this study, it was found that bHLH93 significantly activated DFR, an important gene in anthocyanin synthesis pathway (Fig. 8). Previous studies have shown that low temperature stress can promote anthocyanin accumulation while affecting plant growth and development [47], bHLH93 may be the bridge connecting anthocyanin pathway initiation under low temperature stress.

In this study, the results of the Y1H and dual luciferase assays demonstrated that MYB3 can bind to the *DFR* promoter in vitro, whereas ERF23, MYB3 and bHLH93 can bind to the *ANS2* promoter. However, no significant activation or inhibition effect was identified in tobacco leaves. This may due to the fact that the in-vivo performance of many TFs is affected by other cofactors, non-coding RNAs or signals from pathways that are induced by environmental conditions [48, 49]. In previous studies, ERF, MYB and bHLH TFs were found to require the formation transcription complexes to perform their regulatory functions [50]. It was reported that CmMYB4 in chrysanthemum (*Chrysanthemum morifolium*) can bind to the *CmbHLH2* promoter, but it requires recruitment of a corepressor, CmTPL, to inhibit the expression of *CmbHLH2* [51]. In strawberry (*Fragaria vesca*), FvbHLH9 is a light-inducible TF which can specifically bind to the promoter of *FvDFR*, and it exerts its activating function by binding with FvHY5 under light conditions [52]. Moreover, during plant growth and development, expression regulation by TFs may occur only in specific tissues or at specific developmental stages [53, 54]. DNA structure or chromatin state can also influence the binding of TFs to promoters and consequently, the expression of downstream genes [55, 56]. Last but not least, biological false-positive Y1H results cannot be fully excluded by biological replications. Future results from transformation validation in grapes will provide us with new information on the regulation of *DFR* and *ANS2* promoters by the suggested TFs.

### Other regulatory factors in flavonoid accumulation

Flavonoids are synthesized in the cytoplasm and then transported to vacuoles for storage or to other destinations where they function as bioactive molecules. Different compartments for flavonoid biosynthesis, storage and function require effective transport mechanisms to realize their biological functions. In grape, VvABCC1, VvGST4, AM1 and AM3 have confirmed roles in anthocyanin transport, and VvGST1 in proanthocyanidin transport [57]. VvMATE1/2, which are homologs of AtTT12 in *Arabidopsis thaliana*, are located in vacuoles and the Golgi complex, respectively [58]. Stress-induced TaGSTL1 selectively recognizes flavonol in wheat [59]. Our integrated DEG and DAM analysis suggested that GST14 and MATE4 play a role in flavonoid transport, while GST8 might be involved in the transport of quercetin glucoside, hesperetin glucoside and epicatechin-epiafzelechin. Compared to the skin, the expression of transporter genes in the flesh was lower; inefficient transport after synthesis could be another reason for the lower accumulation of flavonol in the flesh.

Structural and regulatory genes in the flavonoid-biosynthesis pathway can be induced by light [60]. Light-induced anthocyanin accumulation requires light-responsive elements, including Constitutively Photomorphogenic 1 (COP1) and Long Hypocotyl 5 (HY5) [61]. In apple, MdMYB1 accumulates in the light and is degraded in the dark [62]. In addition, under low ultraviolet irradiation, the transcription levels of *FLS*, *HY5* and *MYB10* in apple fruit are downregulated, and anthocyanin and flavonol contents decrease [63]. In pear, PpBBX16 is an activator of light-induced anthocyanin accumulation [64]. In nature, in many red-fleshed fruit, flesh coloration does not depend on light signals, and follows a different timeline from the light-dependent skin coloration, such as in fig (*Ficus carica* L.) [65]. Anthocyanin accumulation in the flesh of ‘Mio Red’ berry occurs in little or no light; further elucidation of this mechanism could help us improve the coloring of this high economic value fruit.

## Conclusion

The dark-skinned table grape variety ‘Mio Red’ develops flesh coloration at ripening, due to anthocyanin accumulation. Metabolome analysis revealed a different spectrum of flavonoids in the flesh and skin, with pelargonidin derivatives and peonidin derivatives as the major anthocyanins in the former. Transcriptome analysis revealed preferential expression of *ANS* and differential expression of a small number of MYB TFs in the flesh and skin, including both activators and repressors. The results of the candidate TFs validation assays showed that MYBA1 in the ‘Mio Red’ grape can promote the biosynthesis of anthocyanins in the flesh. Meanwhile, ERF23 and ERFCBF6 participate in the regulation of anthocyanin synthesis through an interaction network. The present study proved our hypothesis that flesh coloration is an independently regulated event in grape berry, and use of the recruited genes for further mechanism elucidation will help pave the way for breeding seedless red-fleshed table grapes with the health benefits of red wines.

## Materials and methods

### Plant material

‘Mio Red’ vines were grown at Shangzhuang experimental station of China Agricultural University, Beijing (40°23ʹN, 116°49ʹW). Onset of anthesis was on 20 May 2020. From June to August, five samples were collected: 20 days after anthesis (DAA, Stage I on the double-sigmoid curve of fruit development), 35 DAA (end of Stage I), 65 DAA (late Stage II, before veraison), 72 DAA (middle of stage III) and 86 DAA (late stage III). Each sampling consisted of three biological replicates, 40 berries per replicate. Samples were taken randomly from eight clusters growing on different vines, and transported to the laboratory on ice. The berry skin was carefully separated with a scalpel, then the skin and flesh were quickly frozen in liquid nitrogen and stored separately at -80 °C for further use. Based on the flesh color at the five sampling time points, the grape berries collected 86 DAA were selected for metabolomic and transcriptomic analyses.

### Determination of total anthocyanin

Tissue anthocyanin content was measured following Ni et al. [66]., with some modifications. Briefly, ~ 0.2 g grape tissue was ground in liquid nitrogen, then mixed with 1 mL methanol: acetic acid solution (99:1, v/v), and placed in a refrigerator at 4 °C for 24 h. The absorbance of the supernatant at 530, 620 and 650 nm was measured by ultraviolet spectrophotometer (Beckman Coulter, Brea, CA, USA). The anthocyanin content was calculated as: [(A_530_ - A_650_) − 0.2 × A_650_ - A_620_].

### Determination of total content of flavonoids

Flavonoid content was determined as in Robinson et al. [67]. After the sample was thoroughly ground in liquid nitrogen, ~ 0.2 g was mixed with 1 mL precooled 80% ethanol, and extracted at 4 °C for 24 h. After centrifuging at 12,000 rpm at 4 °C for 20 min, 0.5 mL supernatant was transferred to a 10-mL centrifuge tube, then 0.3 mL 8% NaNO_2_, 0.3 mL 10% Al(NO_3_)_3_ solution, 2 mL 2 M NaOH solution and 4.9 mL ethanol were added consecutively. After standing for 10 min, the absorbance of the reaction solution was determined at 510 nm. Rutin was used as the standard, and the final flavonoid content was calculated as mg rutin/g fresh weight.

### Flavonoid metabolome analysis

Flavonoid metabolome analysis was carried out by Metware Biological Science and Technology Company Ltd. (Wuhan, China) using a well-established protocol [68, 69]. Briefly, ~ 1 g of the frozen-stored grape flesh/skin samples was vacuum freeze-dried, then ground to a fine powder with a mixer mill (MM 400, Retsch). For each extraction, 100 mg powder was dissolved in 1 mL 70% methanol, left to stand at 4 °C for 24 h, then centrifuged at 10,000* g* for 10 min. The supernatant was filtered through a microporous membrane (0.22 μm) for LC-MS/MS analysis. Each sample consisted of three biological replicates.

Analytical conditions for HPLC were as follows. We used a Waters ACQUITY UPLC HSS T3 C18 column (1.8 μm, 2.1 mm x 100 mm). The solvent system consisted of solution A (0.04% acetic acid in water) and solution B (0.04% acetic acid in acetonitrile), with gradient program 100 A:0B (v/v) at 0 min, 5 A:95B at 11.0 min, hold for 1 min, 95 A:5B at 12.1 min, hold to 15.0 min; flow rate was 0.40 mL/min. The column temperature was 40 °C, and the injection volume was 2 µL.

The effluent was connected to a triple quadrupole-linear ion trap (Q TRAP) mass spectrometer equipped with a dual electrospray ionization source in positive ion mode and controlled by Analyst 1.5 software (AB Sciex, Ontario, Canada) [60]. Instrument tuning and mass calibration were set with 10 and 100 µmol/L polypropylene glycol solutions in QQQ and LIT modes, respectively. Purine (C_5_H_4_N_4_) and HP-921 [hexakis-(1 H, 2 H, 3 H-terrafluoro-pentoxy) phosphazene] (C_18_H_18_O_6_N_3_P_3_F_24_) were added for internal mass calibration. According to the metabolites eluted in each period, and the specific set of Q1 and Q3 data, metabolites were identified by annotation against the commonly used Metware Data Base [70]. Thresholds of VIP (variable impact in project) ≥ 1 and fold change ≥ 2 or ≤ 0. 5 were set as criteria for metabolites with significant differences.

### Transcriptome analysis

‘Mio Red’ grape skin and flesh collected 86 DAA were selected for total RNA extraction using a modified CTAB method [71], with three biological replicates per sample. RNA quality was checked by 1% agarose gel electrophoresis, a NanoPhotometer spectrophotometer (NanoDrop Technologies, Wilmington, DE, USA), a Qubit 2.0 fluorometer (Life Technologies, Thermo Fisher Scientific, Waltham, MA, USA), and an Agilent 2100 Bioanalyzer (Agilent Technologies, Palo Alto, CA, USA) before constructing the libraries. The mRNA enrichment and cDNA library construction followed standard protocols. The libraries were sequenced on an Illumina HiSeq platform after qualified quality inspection. After removing the sequencing connectors and low-quality reads, the clean reads were annotated against the grape reference genome [72] and the libraries were compared using HISAT2 [73]. Fragments per kilobase of exon per million fragments mapped (FPKM) was used to measure the expression level of the transcripts. DESEQ2 [74] was used to analyze the differential expression among sample groups. The differentially expressed genes (DEGs) were screened with |log_2_fold change| ≥ 1 and FDR < 0.05.

### Quantitative real-time PCR (qRT-PCR)

Based on the transcriptome data, 11 structural genes in the flavonoid-biosynthesis pathway were selected, and their expression levels at the five sampling timepoints of berry development were validated by qRT-PCR. The primers are listed in Table S1. The PCR was carried out with ABI QuantStudio 6 (Applied Biosystems) using ChamQ Universal SYBR qPCR Master Mix (Vazyme, Nanjing, China). The 10-µL amplification system consisted of 5 µL SYBR qPCR Master Mix, 0.2 µL upstream and downstream primers (10 µM each), 1 µL template, and 3.6 µL distilled water. The amplification procedure was 95 °C 30 s; 40 cycles of 95 °C 10 s, 60 °C 30 s; 95 °C 15 s; 60 °C 60 s; 95 °C 15 s. All samples were run in at least three technical repetitions. Grape *β-actin* was used as the reference gene, and the data were quantitatively analyzed by 2^−ΔΔCT^ method. Means and standard errors (SEs) were calculated using one-way analysis of variance (ANOVA) followed by Duncan’s test with SPSS Version 16.0 (Chicago, IL, USA). The significance level was set to *p* < 0.05.

### Yeast one-hybrid assay

Y1H assay was performed according to the Yeast Laboratory Manual (Clontech, USA). Vectors pBait-AbAi and pGADT7 were used. The promoter region of *VvDFR* / *VvANS2* was cloned, and *cis*-acting regulatory elements were predicted by PlantCARE (http://bioinformatics.psb.ugent.be/webtools/plantcare/html/). The promoter region and the full-length TF sequences were inserted to the pBait-AbAi and pGADT7 vectors respectively (primer sequences in Table S1). The one-to-one interaction verification was carried out after the self-activation of yeast was inhibited by designing different AbA concentrations. The specific operation was followed Wang et al. [75].

#### Dual luciferase assay

The dual luciferase assay was according to Wu et al. [76]. The candidate TFs and promoter region of *VvDFR* / *VvANS2* were constructed into the vector pGreenII 62-SK and pGreenII 0800-LUC (with Firefly luciferase reporter gene), respectively. The primers for all the constructed vectors are listed as Table S1. All the constructed vectors were transformed into *Agrobacterium* strain GV3101(pSoup). *Nicotina benthamiana* leaves were selected as the transiently expressed plant material. The enzyme activities of firefly luciferase (LUC) and Renilla luciferase (REN) were detected using the Dual-Luciferase Reporter Assay System and Modulus Luminometer (Promega, USA). The LUC /REN value of TFs on *VvDFR* / *VvANS2* was calculated by normalization to reflect the regulatory effect of candidate TFs.

## Electronic supplementary material

Below is the link to the electronic supplementary material.

Supplementary Material 1: Table S1: Primer sequences for genes used in assays in this study. Table S2: List of metabolites detected and quantified in the skin and flesh of ‘Mio Red’ grape. Table S3: List of metabolites differentially accumulated in the skin and flesh of ‘Mio red’ grape. Table S4: Relative contents of the 6 anthocyanin derivatives identified in the skin and flesh of ‘Mio Red’ grape. Table S5: Quality check of the raw data from the RNA-seq. Table S6: Overview of transcriptome sequencing dataset and mapping statistics of ‘Mio Red’ grape. Table S7: Expression profile (FPKM values) of the 23,786 genes detected in the skin and flesh of ‘Mio Red’ grape. Table S8: Identification of structural genes in flavonoid-biosynthesis pathway of ‘Mio Red’ grape. Table S9: GenBank accession numbers of the transcription factors in the phylogenetic tree analysis.

Supplementary Material 2: Fig. S1 Flavonoid metabolomes in the skin and flesh of ‘Mio Red’. (a) Correlation diagram between samples. (b) Principal component analysis score chart based on mass spectrum data of samples. Mix indicates control sample. (c) Hierarchical clustering heatmap based on flavonoid metabolite profiles in the skin and flesh of ‘Mio Red’ grape. Fig. S2: (a) Volcano plots of differentially abundant metabolites between ‘Mio Red’ grape skin and flesh. (b) KEGG enrichment of the differentially abundant metabolites. Fig. S3: Network of flavonoid-related genes (blue) and metabolites (red) in ‘Mio Red’ grape. There are 4 KEGG pathways: flavonoid biosynthesis, flavone and flavonol biosynthesis, anthocyanin biosynthesis and isoflavone biosynthesis. Fig. S4: Dual luciferase (LUC) assay of MYB3 and promoter of *VvDFR* and *VvANS2*.

## Data Availability

The raw RNA-seq data are available at: https://www.ncbi.nlm.nih.gov/bioproject/PRJNA781680.

## Abbreviations

ANS: Anthocyanin synthase
UFGT: UDP-glucose:flavonoid-3-*O*-glucosyltransferase
bHLH: Basic helix-loop-helix
ERF: Ethylene response factor
DAMs: Differentially accumulated metabolites
KEGG: Kyoto encyclopedia of genes and genomes
DEGs: Differentially expressed genes
CHS: Chalcone synthase
CHI: Chalcone isomerase
F3H: Flavanone 3-hydroxylase
F3’H: Flavanone 3′-hydroxylase
DFR: Dihydroflavonol 4-reductase
F3’5’H: Flavonoid 3′,5′-hydroxylase
FPKM: Fragments per kilobase of exon model per million mapped reads
FLS: Flavonol synthase
ANR: Anthocyanidin reductase
LAR: Leucoanthocyanidin reductase
Y1H: Yeast one-hybrid
TF: Transcription factor
