# Supplementary figures and images for: Independent flavonoid and anthocyanin biosynthesis in the flesh of a red-fleshed table grape revealed by metabolome and transcriptome co-analysis
Source: BMC Plant Biol. 2023 Jul 15;23:361. doi: 10.1186/s12870-023-04368-8 (PMC10349436; doi:10.1186/s12870-023-04368-8)

## Slide 1
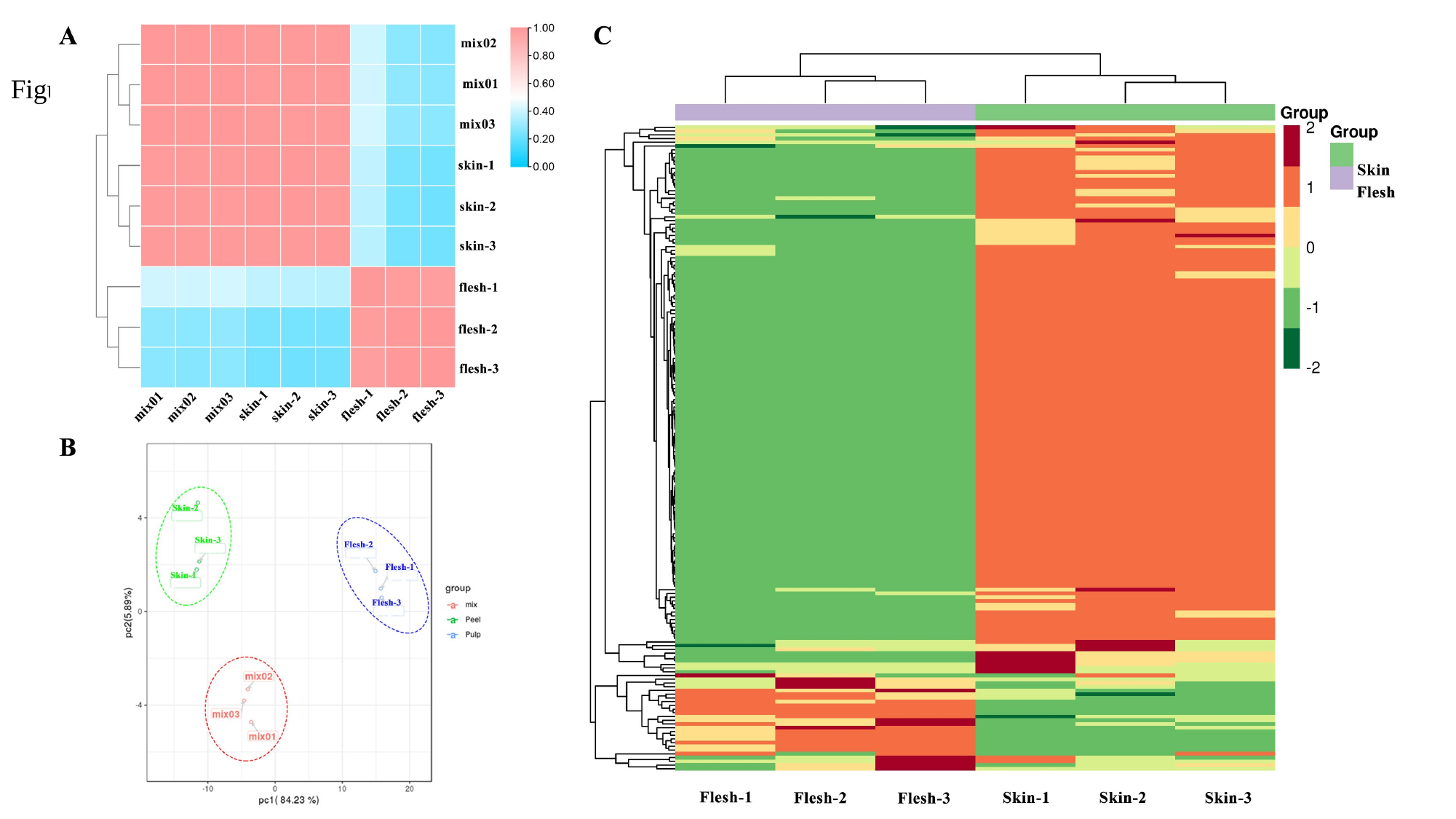

Figure S1

## Slide 2
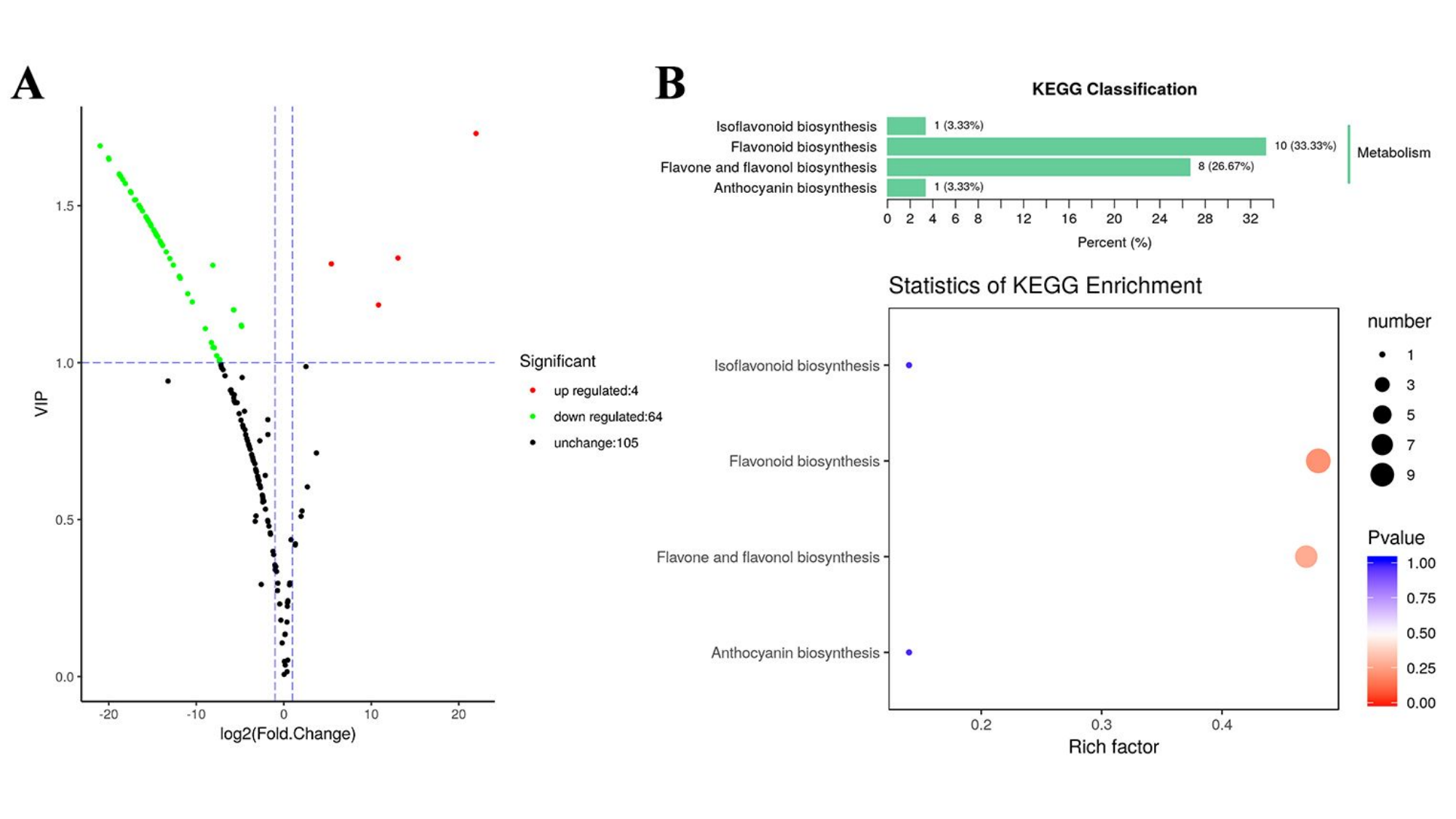

Figure S2

## Slide 3
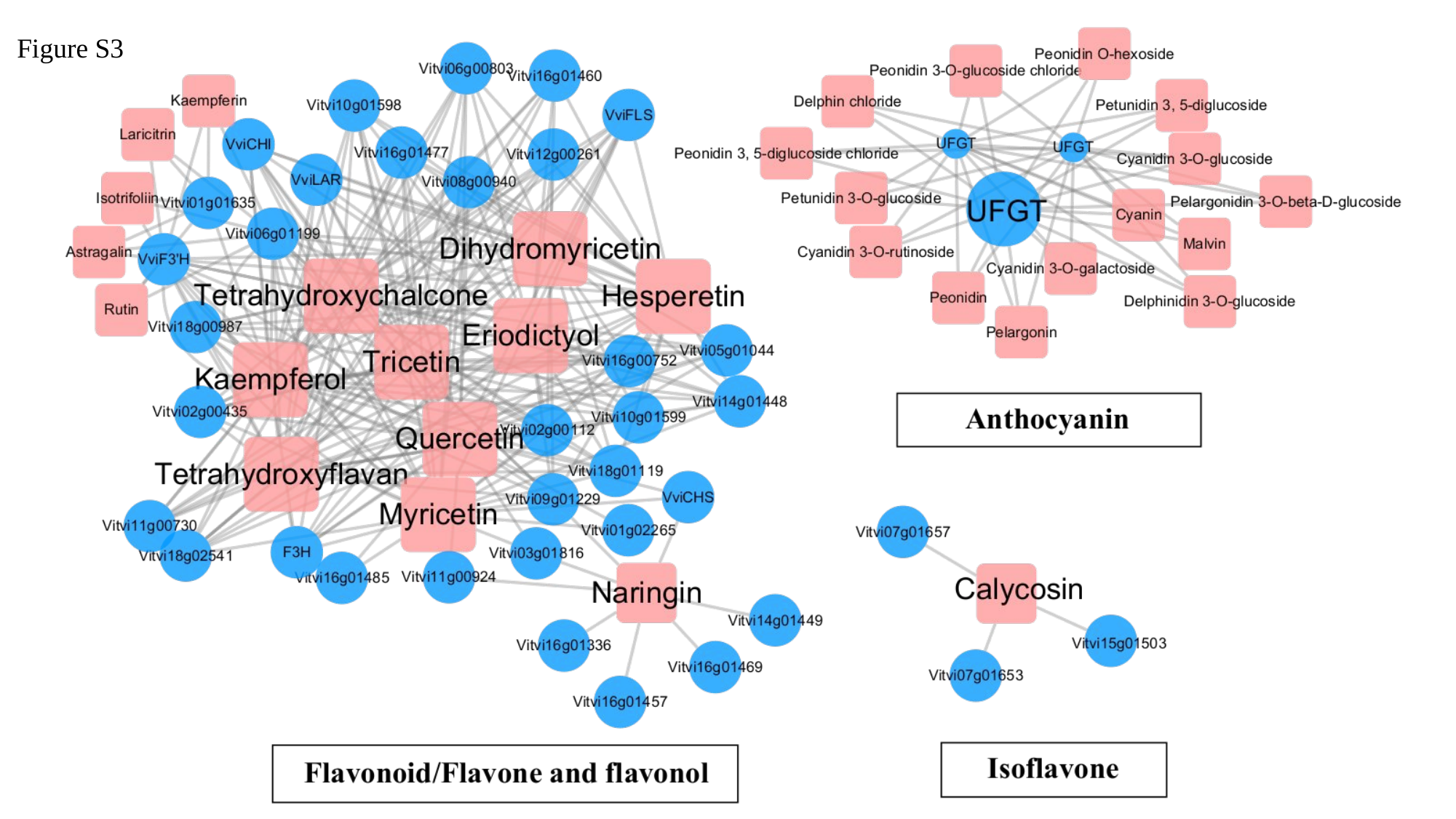

Figure S3

## Slide 4
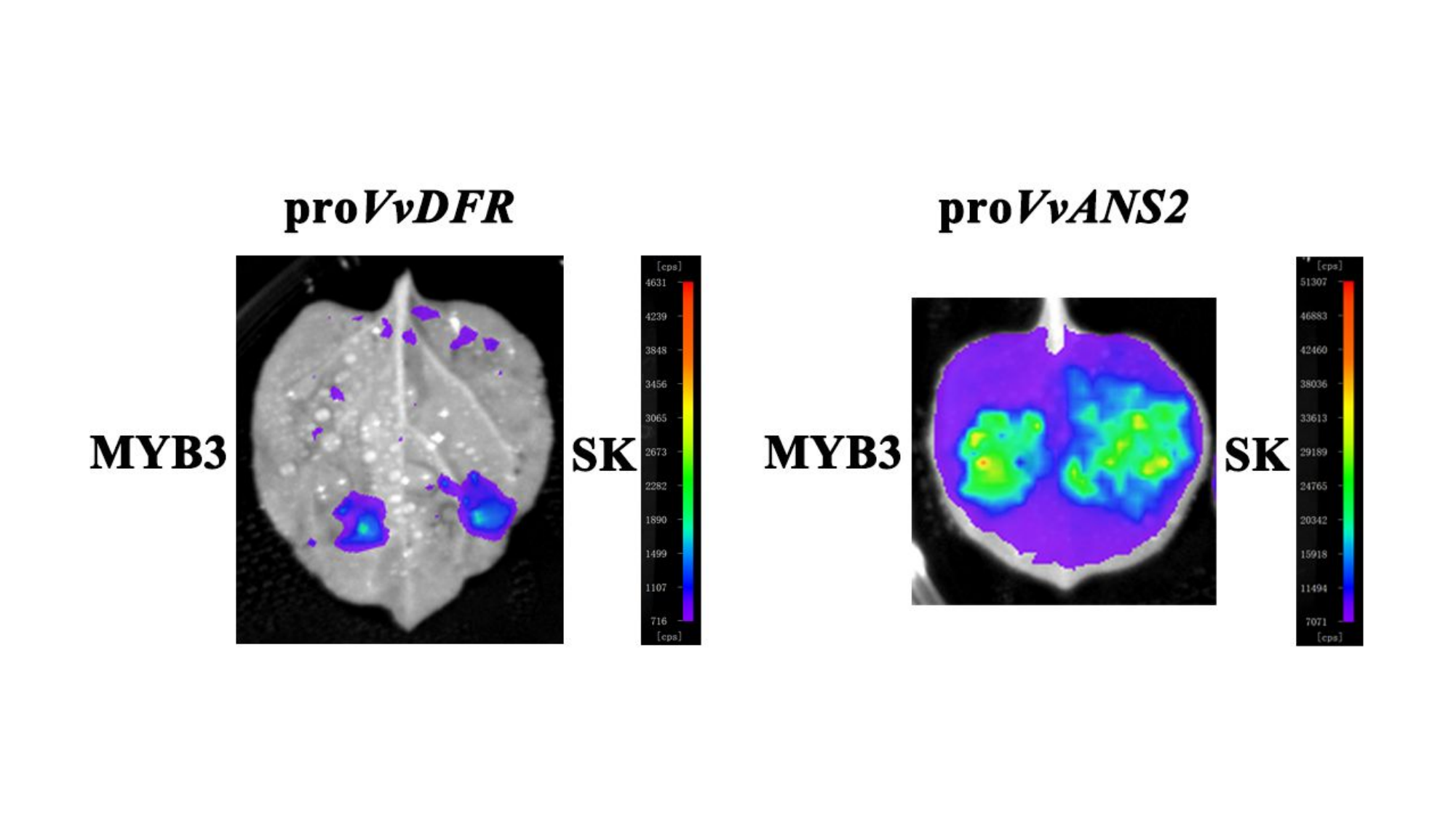

Supplement: Supplementary file 2 — Supplementary Material 2: Fig. S1 Flavonoid metabolomes in the skin and flesh of ‘Mio Red’. (a) Correlation diagram between samples. (b) Principal component analysis score chart based on mass spectrum data of samples. Mix indicates control sample. (c) Hierarchical clustering heatmap based on flavonoid metabolite profiles in the skin and flesh of ‘Mio Red’ grape. Fig. S2: (a) Volcano plots of differentially abundant metabolites between ‘Mio Red’ grape skin and flesh. (b) KEGG enrichment of the differentially abundant metabolites. Fig. S3: Network of flavonoid-related genes (blue) and metabolites (red) in ‘Mio Red’ grape. There are 4 KEGG pathways: flavonoid biosynthesis, flavone and flavonol biosynthesis, anthocyanin biosynthesis and isoflavone biosynthesis. Fig. S4: Dual luciferase (LUC) assay of MYB3 and promoter of VvDFR and VvANS2. [file 12870_2023_4368_MOESM2_ESM.pptx]
